# Follow-up study of the cardiac manifestations of multiethnic patients hospitalised with COVID-19

**DOI:** 10.1007/s40477-026-01193-1

**Published:** 2026-07-30

**Authors:** Gabriel Bioh, Ashish Patel, Reinette Hampson, Emma Howard, Roxy Senior

**Affiliations:** 1 Department of Cardiology, Northwick Park Hospital, Harrow, HA1 3UJ UK; 2 Department of Cardiology, Royal Brompton Hospital, London, SW3 6NP UK; 3 National Heart and Lung Institute, Imperial College, London, UK

**Keywords:** COVID-19, Echocardiography, Follow-up, Multiethnic

## Abstract

**Purpose:**

To determine the natural history of the cardiac manifestations, determined by advanced transthoracic echocardiography (TTE), due to COVID-19 in multiethnic hospitalised patients.

**Methods:**

A prospective observational study, in a London NHS Trust, of patients admitted with COVID-19 undergoing TTE. Baseline TTE was taken during acute admission with follow-up TTE at a median of 17.6 months to assess the same left ventricular (LV), right ventricular (RV) and pulmonary pressure parameters with additional 3D and strain measures.

**Results:**

There were a total of 117 patients with admission and follow-up TTE. LV parameters were largely unchanged at follow-up except E/A ratio decreasing from 0.98 to 0.85. LV global longitudinal strain measures detected subclinical LV impairment in 36% of patients. RV dimensions improved (mean mid RV diameter, 3.1 cm to 2.7 cm (*p* < 0.001)) but with persistence of RV impairment at follow-up (28%). 3D RV ejection fraction and global strain measures determined impairment in around 40% of patients. All pulmonary haemodynamic measures showed improvement with resolution of effects of RV afterload (*p* = 0.016), decrease in pulmonary artery systolic pressure (36 mmHg to 28 mmHg *p* = 0.032) and improvements in mean pulmonary valve acceleration time (113 ms to 120 ms), *p* = 0.025, and pulmonary vascular resistance (2.2 to 1.7 WU), *p* = 0.01.

**Conclusion:**

Follow-up echocardiographic parameters compared to admission parameters of multiethnic hospitalised patients with COVID-19 demonstrate improved pulmonary haemodynamics but persistent LV and RV impairment which suggest possible myocarditic/microthrombotic lasting effects of COVID-19 infection.

**Supplementary Information:**

The online version contains supplementary material available at https://doi.org/10.1007/s40477-026-01193-1.

## Introduction

From the onset of the COVID-19 pandemic, there was an early signal of an effect on the heart, manifesting in a raised Troponin above the 99th percentile as well as raised Brain natriuretic protein (BNP) [1, 2 ] and this in turn was associated with a poorer prognosis in the form of intensive care unit (ICU) admission and mortality [3–6]. Such elevated biomarkers reflected impairment of left ventricular (LV) as well as right ventricular (RV) function [7–11], although the exact mechanism was unclear and is currently thought to be multifactorial including a myocarditic process, myocardial infarction through exacerbation of underlying atherosclerotic disease as well as through a microthrombotic process [12–14]. In the setting of myocardial damage, it is important to know the permanence or reversibility of such damage. Data to date have been conflicting as to the longer term outcomes of the cardiac effects of COVID-19. Some studies have suggested that there is deterioration in left and right heart function [15] while others suggest improvement in left and right heart function [16] and others, suggest a combination of the two with improvement in right ventricular parameters but no improvement in left ventricular parameters [17, 18 ]. Further clarity on the longer term cardiac manifestations of COVID-19 infection is therefore warranted. Thus, we prospectively looked at the acute cardiac manifestations in hospitalised multiethnic COVID-19 patients [10] as well as follow-up of survivors to determine persistence, worsening or resolution of disease.

## Material and methods

### Enrolment

Patients within the London North West University Healthcare NHS Trust referred for transthoracic echocardiography (TTE) for suspected cardiac pathology due to COVID-19 were prospectively enrolled from March 1st 2020 to August 19th 2021. COVID-19 positivity was determined by positive COVID-19 polymerase chain reaction (PCR) swab or a chest radiograph or computed tomography (CT) scan of the chest demonstrating probable COVID-19 in the context of symptoms and signs consistent with COVID-19. Patients were followed up with repeat TTE at a minimum of 6 months post admission (with the exception of 2 patients, 1 of whom had a repeat TTE at 4.8 months post admission and the second at 5.5 months post admission). The data was collected as part of a prospective study reviewed and approved by Trust R&D and local research ethics committee. Individual consent was obtained for inclusion of data in the study.

### Patient and public involvement

The emergency circumstances of COVID-19 precluded patient or public involvement in the design, conduct or reporting of this study.

### Echocardiography

Follow up TTE was performed by the experienced research team of cardiac physiologists and fellow (AP, RH and GB) and then reviewed by the head cardiologist (RS). Admission TTE was performed by experienced cardiac physiologists and fellows (including but not exclusively by AP and GB) and all were reviewed by the head cardiologist (RS). Measurements were taken as per American Society of Echocardiography and European Association of Cardiovascular imaging recommendations for cardiac chamber quantification in adults [19]. A full dataset was taken to include assessment of LV dimension and function with Simpson’s biplane, 3D assessment and strain for global longitudinal strain (GLS). RV size and function were assessed, specifically measuring tricuspid annular plane systolic excursion (TAPSE), RV S’ velocity on tissue Doppler imaging (RVS’TDI), RV fractional area change (RVFAC), right ventricular outflow tract velocity time integral (RVOT VTI), RV free wall longitudinal strain (RVFWLS), RV global longitudinal strain (RVGLS) and 3D RV ejection fraction (3D RVEF). Pulmonary measures included pulmonary valve acceleration time (PVAT), estimation of pulmonary artery systolic pressure (PASP) using tricuspid regurgitation to estimate pressure difference across the tricuspid valve and adding the estimated right atrial pressure (RAP), mean pulmonary artery pressure (mPAP) using peak pulmonary regurgitation velocity to estimate pressure gradient and adding estimated RAP, and pulmonary vascular resistance (PVR) measured using the peak tricuspid regurgitant velocity (m/s) and dividing this by the RVOT VTI (cm), multiplying the result by 10 and adding a constant 0.16. Left atrial assessment included left atrial reservoir strain, left atrial conduit strain, left atrial contraction strain and left atrial volume indexed to body surface area. All studies were conducted on an EPIQ CVx ultrasound system (Philips Healthcare Eindhoven, Holland).

### Statistical analysis

The IBM SPSS statistics 28 package was used for all statistical analysis. In all analyses, P values of less than 0.05 were considered statistically significant. Normally distributed independent continuous data was analysed using the Student t-test, and paired continuous data with the paired-samples t-test. Normality was assessed using quantile–quantile plots and the Shapiro–Wilk test. Non-normally distributed independent continuous data was analysed using the Mann–Whitney U Test and paired continuous data by the Wilcoxon signed rank test.

Categorical independent data were analysed using the Chi-Square Test of Independence, however, where expected frequencies were less than 5 in 20% or more of cells, or sample size was less than 40, Fisher’s Exact Test was used for analysis. For categorical data of repeated measures with two categories of response, McNemar’s test was used.

Given the large number of echocardiographic parameters analysed across multiple cardiac domains, a False Discovery Rate (FDR) adjustment set to 5% was applied to reduce the probability of making a type I statistical error.

## Results

**Fig. 1:**
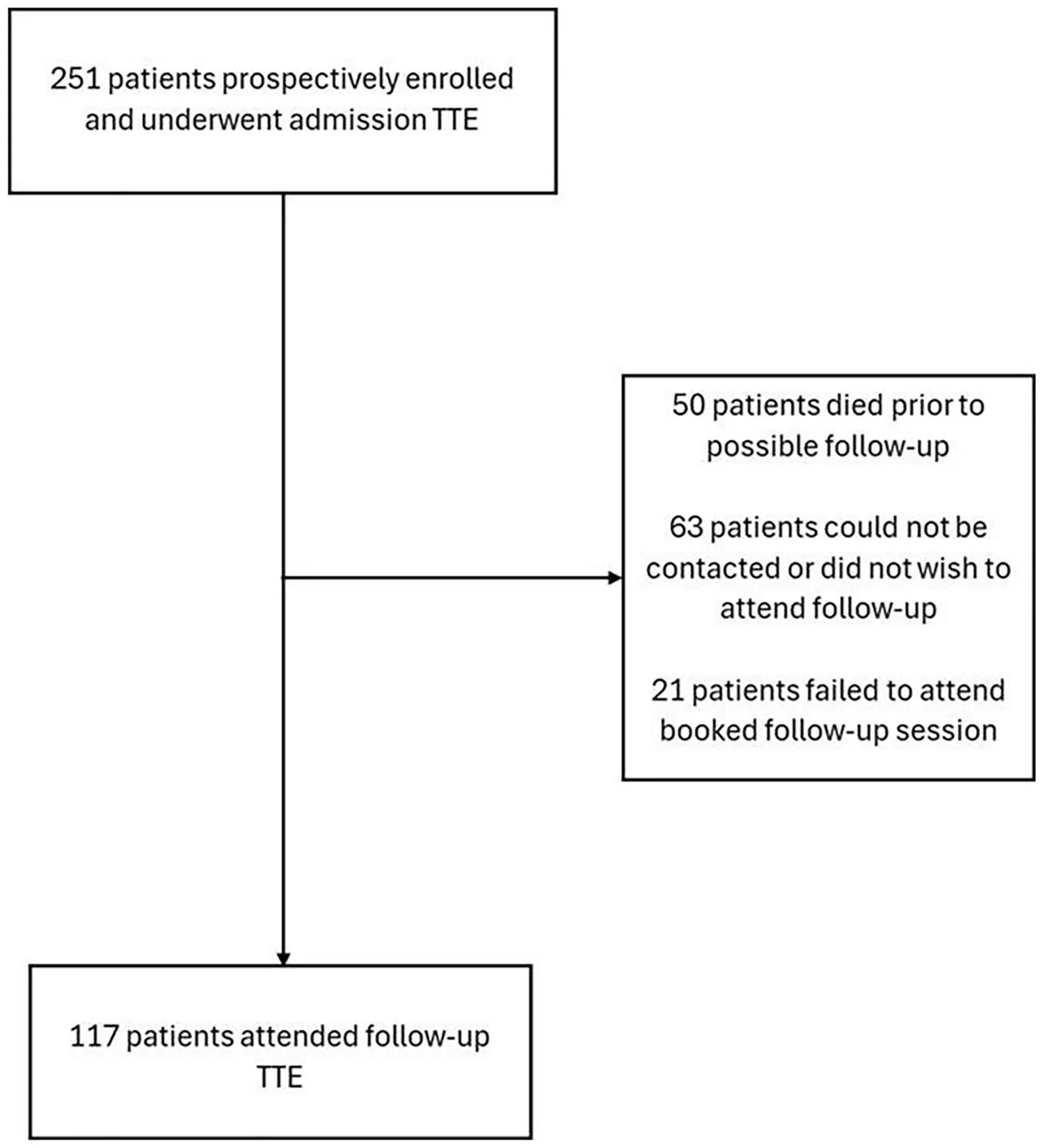
Flow chart demonstrating selection of patients for follow-up transthoracic echocardiogram (TTE)

From an original total of 251 patients consented at the index admission for COVID-19, a total of 117 patients were available for follow-up echocardiography. Reasons for patients being unavailable for follow-up echocardiogram included 1) death, 2) wish not to participate in follow-up TTE, 3) not being contactable to book follow-up TTE and 4) failure to attend follow-up TTE once booked (Fig. 1). The follow-up TTE occurred at a median duration of 17.6 months after the original TTE from acute admission, with a minimum of 4.8 months and maximum of 27.7 months.

### Patient demography

Table 1 shows the patient demography and co-morbid conditions of the patient population studied. The mean age of patients was 59.4 ± 12.8 years, 73% were male, 15% were white and the mean BMI was 29.3 ± 6.9 kg/m^2^. Systemic hypertension was the most common co-morbid condition, present in 51% of patients, followed by diabetes mellitus, present in 37% and then cardiovascular disease (known ischaemic heart disease, cardiomyopathy, significant valvulopathy or arrhythmia) in 15% of patients and 9% had chronic kidney disease. Ethnicities were defined for 103 out of 117 patients. The breakdown is as follows: White 15 (15%), Black (British, African, Caribbean and other) 13 (13%), Asian (Indian, Pakistani, Sri Lankan and Bangladeshi) 36 (35%), Asian (Chinese and other) 28 (27%), Mixed 2 (2%), Other 9 (9%).

**Table 1:** Table of recorded variables for patient demography and co-morbid conditions

Variable	Follow-up patients (117)
*Demographics*	
Age at admission, years, mean (± SD)	59.4 (± 12.8) [117]
Gender, male, number (%)	85 (73%) [117]
Ethnicity, white, number (%)	15 (15%) [103]
BMI (kg/m^2^), mean, (± SD)	29.3 (± 6.9) [109]
*Co-morbid conditions*	
Hypertension, number (%)	60 (51%) [117]
Diabetes, number (%)	43 (37%) [117]
Cardiovascular disease, number (%)	17 (15%) [117]
Cerebrovascular disease, number (%)	8 (7%) [117]
Cancer, number (%)	5 (4%) [117]
CKD, number (%)	11 (9%) [117]
Respiratory disease, number (%)	28 (24%) [117]

Means and standard deviations displayed for continuous variables and number and percentage for categorical variables. Number in square bracket records total number of patients.

### Echocardiographic features from acute admission vs follow-up

#### Left ventricle assessment (Table 2)

**Table 2:** Table of recorded variables for the left ventricle. The variables were compared between the TTE at admission and the follow-up TTE

Variable	Echo during admission (117)	Echo at follow-up (117)	*P* value
*Left heart parameters*			
LV end-systolic dimension (cm) mean (± SD)	2.8 (± 0.6) [115]	2.9 (± 0.6) [115]	0.094
LV end-diastolic dimension (cm) mean (± SD)	4.4 (± 0.6) [115]	4.6 (± 0.6) [117]	**0.006**
LA vol index (ml/m^2^), mean (± SD)	27.7 (± 11.6) [70]	26.7 (± 10.0) [109]	0.642
E/A, mean (± SD)	0.98 (± 0.4) [106]	0.85 (± 0.3) [111]	** < 0.001**
E/e' average, mean (± SD)	10.1 (± 4.2) [109]	9.2 (± 3.4) [114]	0.060
E/e' average > 14, number (%)	18 (17%) [109]	10 (9%) [114]	0.065
New elevated LV filling pressure + Normal *LVEF > 50%, number (%)*	4 (6%) [72]	7 (6%) [114]	1.000
New LV RWMA, number (%)	1 (1%) [69]	5 (5%) [104]	0.250
Abnormal LVEF (< 50%), number (%)	8 (7%) [117]	8 (7%) [117]	1.000
3D LVEF (%), mean (± SD)		56.4% (± 6.5) [79]	
3D LVEF < 50%, number (%)		8 (10%) [79]	
LV GLS (%), mean (± SD)		18.4 (± 2.8) [89]	
LV GLS < 18%, number (%)		32 (36%) [89]	
LA Reservoir strain (%), mean (± SD)		32.9 (± 11.2) [100]	
LA Reservoir strain < 18%, number (%)		10 (10%) [100]	

Means and standard deviations displayed for continuous variables and number and percentage for categorical variables. Number in square bracket records total number of patients. Significant p values are in bold.

When considering left ventricular parameters, there was no significant difference between indexed LV end-diastolic and end-systolic dimensions from admission to follow-up. With regards to left ventricular function, there was no net difference between admission and follow-up numbers of patients with LV systolic impairment (noted to be 8 out of 117 (7%) in both cases). Of the 8 patients with abnormal LV systolic function (LV ejection fraction (LVEF) < 50%) at admission, 4 improved to normal while the other 4 did not change. However, at follow-up 4 new patients developed abnormal LV systolic function. Generally, LV filling pressure as assessed by E/A ratio and E/e’ showed improvement at follow-up, however, there was persistence of impairment of LV relaxation. When assessing diastolic heart failure as defined by newly elevated LV filling pressure with normal LVEF ≥ 50%, there was no difference between admission and follow-up, (6%). Prevalence of regional wall motion abnormalities was low at baseline and follow-up. A more detailed echocardiogram was conducted at follow-up utilising 3D and global longitudinal strain techniques where image quality was sufficient. Mean 3D LVEF was 56.4% with impairment in 8 out of 79 patients (10%) defined as LVEF < 50%. LV GLS was 18.4% on average with 32 out of 89 patients (36%) having impaired GLS < 18% [20]. Mean LA reservoir strain was 32.9% from 100 patients and was reduced (< 18%) [20] in 10 out of 100 patients (10%).

#### Right ventricle assessment (Table 3)

**Table 3:** Table of recorded variables for the right ventricle. The variables were compared between the TTE at admission and the follow-up TTE

Variable	Echo during admission (117)	Echo at follow-up (117)	*P* value
*Right heart parameters*			
RV impairment, number (%)	33 (28%) [117]	33 (28%) [116]	1.000
TAPSE (cm), mean (± SD)	2.2 (± 5.3) [60]	2.0 (± 0.4) [110]	0.145
Reduced TAPSE < 1.7 cm, number (%)	8 (13%) [60]	14 (13%) [110]	1.000
FAC (%), mean (± SD)	40.0 (± 11.2) [66]	39.6 (± 8.6) [105]	0.475
Reduced FAC < 35%, number (%)	19 (29%) [66]	27 (26%) [105]	1.000
RV S’ TDI (cm/s), mean (± SD)	14.8 (± 3.8) [110]	13.2(± 3.3) [110]	** < 0.001**
Basal RV diameter (cm), mean (± SD)	3.7 (± 0.8) [114]	3.3 (± 0.6) [114]	** < 0.001**
Dilated basal RV > 4.1 cm, number (%)	31 (27%) [114]	7 (6%) [114]	** < 0.001**
Mid RV diameter (cm), mean (± SD)	3.1 (± 0.9) [113]	2.7 (± 0.6) [114]	** < 0.001**
Dilated mid RV > 3.5 cm, number (%)	27 (24%) [113]	10 (9%) [115]	**0.005**
RVOT VTI (cm), mean (± SD)	15.3 (± 4.1) [87]	15.2 (± 4.2) [117]	0.479
3D RVEF (%), mean (± SD)		44.7 (± 8.6) [69]	
3D RVEF < 45%, number (%)		29 (42%) [69]	
RV GLS (%), mean (± SD)		18.5 (± 4.6) [96]	
RV GLS < 17%, number (%)		40 (42%) [96]	
RV FWLS (%), mean (± SD)		21.6 (± 5.9) [96]	
RV FWLS < 20%, number (%)		38 (40%) [96]	

Means and standard deviations displayed for continuous variables and number and percentage for categorical variables. Number in square bracket records total number of patients. Significant *p* values are in bold.

RV dimensions showed significant reduction at follow-up. There were reductions of both basal and mid RV dimensions. Parameters measuring RV function, however, did not generally show any significant difference including FAC, TAPSE and RVOT VTI. However, the RV S’ TDI showed a significant reduction from 14.8 cm/s on admission to 13.2 cm/s at follow-up, p < 0.001. The latter’s measurement were more often feasible than TAPSE (110 patients vs 60 patients respectively). Average 3D ejection fraction was 44.7% from 69 patients and abnormal (< 45%) in 29 out of 69 patients (42%). Both RV GLS and RV FWLS were reduced at follow-up. Reduced RV GLS (< 17%) [21] and FWLS (< 20%) [22] were noted in 42% and 40% of patients respectively.

#### Pulmonary haemodynamic assessment (Table 4)

**Table 4:** Table of recorded variables for pulmonary haemodynamics. The variables were compared between the TTE at admission and the follow-up TTE

Variable	Echo during admission (117)	Echo at follow-up (117)	*P* value
*Pulmonary haemodynamics*			
D-shaped LV, number (%)	8 (7%) [113]	0 (0%) [114]	**0.016**
PV acceleration time (msec), mean (± SD)	113 (± 30.4) [88]	120 (± 30.1) [117]	**0.025**
PVAT < 80 ms, number (%)	13 (15%) [88]	7 (6%) [117]	**0.021**
Pulmonary artery systolic pressure (mmHg), mean (± SD)	36 (± 12.7) [55]	28.0 (± 7.8) [34]	**0.032**
Pulmonary vascular resistance (Wood units), mean (± SD)	2.2 (± 0.8) [54]	1.7 (± 0.4) [33]	**0.010**
Elevated pulmonary pressure, number (%)	25 (22%) [114]	8 (7%) [117]	**0.004**
RA filling pressure (mmhg), mean (± SD)	7.1 (± 3.9) [92]	5.1 (± 0.6) [110]	** < 0.01**

Means and standard deviations displayed for continuous variables and number and percentage for categorical variables. Number in square bracket records total number of patients. Significant *p* values are in bold.

All the 8 patients who showed signs of RV ventricular overload (D-shaped LV) on admission showed resolution at follow-up, p = 0.016. Pulmonary valve acceleration time, a measure of pulmonary vascular resistance, showed improvement by follow-up, from 113 to 120 ms, p = 0.025. This is further exemplified by the direct echo measure of pulmonary vascular resistance demonstrating reduction from 2.2 Wood units (WU) to 1.7 WU at follow-up, p = 0.01. Pulmonary artery systolic pressure decreased from a mean of 36 mmHg at admission to 28 mmHg by follow-up, p = 0.032. RA filling pressure was reduced in the follow-up patients, with a mean of 5.1 mmHg compared to 7.1 mmHg on admission. Thus, on admission, 25 out of 114 patients (22%) had elevated pulmonary pressure by at least 1 measure, however, by follow-up only 8 patients out of 117 (7%) had elevated pulmonary pressure, p = 0.004. It should be noted that following FDR adjustment, the continuous variables pulmonary valve acceleration time and pulmonary artery systolic pressure no longer met the threshold for significance. All other variables remained significant.

### Follow-up vs non-follow-up patient characteristics (Table S1, supplementary data)

Comparison of the baseline demographics, biomarkers, comorbid conditions and haemodynamics at admission as well as state of ventilation were compared between the patients who were followed-up (117) and those who we were not (275) to help characterise the difference between these 2 patient populations.

The follow-up patient population was younger than non-follow-up, 59.4 years vs 66.8 years, *p* < 0.001, more predominantly male, 73% vs 62%, *p* = 0.046, with higher BMI, 29.3 kg/m^2^ vs 27.8 kg/m^2^, *p* = 0.020. Of the biomarkers, admission Troponin T and NT-proBNP were significantly lower in the follow-up group with a median of 17 ng/L vs 26 ng/L, *p* = 0.012, and median of 377 ng/L vs 1237 ng/L, *p* < 0.001 respectively. The follow-up group had a lower incidence of cardiovascular disease and cancer, 15% vs 29%, *p* = 0.003 and 4% vs 12%, *p* = 0.018, respectively.

Whilst mean admission heart rate and systolic blood pressure were similar between the 2 groups, admission respiratory rate was higher in the follow-up group at 33 breaths/min compared to 30 breaths/min, *p* = 0.013, and admission saturations on air were lower in the follow-up group at 87% compared to 92%, *p* = 0.022. There was no significant difference in the number of ventilated patients between the 2 groups, *p* = 0.529.

Table S1. Table of recorded variables for demographics, blood biomarkers, comorbid conditions, haemodynamics prior to echocardiogram, and ventilation. Variables for follow-up patients were compared with non-follow-up patients. Means and standard deviations or medians and interquartile ranges are displayed for continuous variables and number and percentage for categorical variables. Number in square bracket records total number of patients. Significant p values are in bold.

### Time to follow-up interaction (Table S2, supplementary Data)

With a follow-up period of up to 27.7 months, it is possible that the length of follow-up in itself could introduce heterogeneity into the analysis as there may be variation in cardiac remodelling during the recovery phase from COVID-19 infection. A bivariate correlation for time to follow-up TTE and each LV, RV and pulmonary parameter demonstrated no significant correlation with maximum correlation coefficient 0.163, p = 0.08. Further, a subgroup analysis was conducted between the earliest half of the data (patients up to and including 535 days follow-up) designated ‘Early follow-up’ versus later half, ‘Late follow-up’. This yielded no significant difference in LV, RV or pulmonary parameter, Table S2.

Table S2. Table of recorded variables for left heart, right heart and pulmonary haemodynamics. Variables for early follow-up patients were compared with late follow-up patients. Means and standard deviations are displayed for continuous variables and number and percentage for categorical variables. Number in square bracket records total number of patients. Significant p values are in bold.

## Discussion

When considering the cardiac effects of COVID-19, we have previously reported a predominant effect on the right ventricle with dilatation and impaired systolic function, predominantly radial. Measures of pulmonary pressure are raised in a significant number of patients but reduction in left ventricular ejection fraction is present but to a lesser extent [10]. Such findings are consistent with other data within the literature [7, 8, 11 ].

Further to this, there have been a number of studies documenting outcomes in patients who have been affected by COVID-19, ranging from mild to severe disease, and utilising either echocardiographic techniques or cardiac MRI. The MRI data has generally been collected in convalescence and compared to control data and has shown varying amounts of scarring consistent with myocarditis as well as scarring due to myocardial infarction and microinfarction [12, 23, 27 –]. In general, whilst demonstrating scar, there is only subclinical effect on LV and RV function. There have also been a number of echocardiographic studies that have considered patients who have recovered from COVID-19 [28–30] suggesting only subclinical LV and RV impairment. However, without direct comparison to imaging during acute admission it is not possible to elucidate the natural history of the disease process within individuals. Where echocardiographic parameters were assessed during hospital admission and compared to follow-up, the data is conflicting, from worsening of LV and RV impairment [15] to improvement in both [16] or improvement in RV impairment but not LV [17, 18 ]. In this study, we were able to follow-up 117 hospitalised patients who had survived COVID-19 infection.

There was a low incidence of left ventricular dysfunction, both systolic (7%) and diastolic (6%), within the study patients of multiethnic origin. At follow-up (median of 17.6 months), there was a similar incidence of left ventricular systolic impairment with some individual patients improving and others deteriorating but no overall net effect. The prevalence of diastolic dysfunction at follow-up was also unchanged. At follow-up, 3D LVEF showed impairment (< 50%) in 10% of patients. However, there was evidence of subclinical impairment of LV function as assessed by global longitudinal strain in 36% of patients at follow-up and abnormal LA reservoir strain in 10%. This is further corroborated by persistence of impaired LV relaxation. Put together, it would appear that there is a subclinical burden of impairment of both systolic (reduced LV GLS) and diastolic function (trend to impaired relaxation and impaired LA reservoir strain) noted in the follow-up population. Without the use of strain at admission for comparison, the initial burden of subclinical disease cannot be determined. A biologically plausible mechanism for persistent subclinical LV impairment includes fibrosis secondary to myocarditis or microthrombotic mediated infarction rather than coronary artery disease per se because of very low prevalence of regional wall motion abnormalities on admission and at follow-up. However, without cardiac MRI data for the patients, this cannot be established.

The RV showed significant reduction in size but with persistent RV impairment in almost 30% of patients. There was no significant improvement in RV radial or longitudinal systolic function as measured by FAC and TAPSE, but rather a reduction in mean RV S’ TDI from 14.8 to 13.2 cm/s, p < 0.001. However, pulmonary haemodynamics showed significant improvement. Features of RV overload resolved accompanied by significant reduction in pulmonary vascular resistance as suggested by increase in mean pulmonary valve acceleration time from 113 to 120 ms with concomitant reduction in calculated pulmonary vascular resistance parameter from 2.2 to 1.7 Wood units. Systolic pulmonary artery pressure decreased from a mean of 36 to 28 mmHg.

Such changes suggest the acute insult to the lungs, mediated by COVID-19 infection, resulted in raised pulmonary pressures and pulmonary vascular resistance leading to RV dilatation. With resolution of the acute infection, pulmonary haemodynamics improved and RV size in turn reduced. Given the load dependent nature of both FAC and TAPSE, it might be expected that with reduction in afterload both measures would improve. This was not the case. TAPSE further suffers from variability in relation to its measure of basal RV function, not reflecting global function and so may not be sensitive to improvement in apical and mid-wall function. The use of 3D RVEF, GLS and FWLS provides a more global assessment of the RV function and the strain measures are less affected by load than the conventional measures of both FAC and TAPSE. The 3D and strain methods confirmed the impairment by conventional measures, demonstrating RV impairment in 40% of patients. This was higher than found with conventional measures (28%) but unsurprising given the superior reflection of global function and lower susceptibility to load highlighted above. Given the lack of 3D and strain data from admission, as with LV and LA, it is not known what proportion of this was preexisting in patients at admission, subclinically.

The fact that RV function did not follow in resolution of dysfunction may well indicate a mechanism other than or in addition to increased right heart pressure mediating impairment. This could be the result of direct damage to the RV most likely in the form of a myocarditic process. This would also explain an element of the LV impairment seen in our study, with scarring as has been seen in cardiac MRI studies. It is also possible that an element of RV impairment was mediated by severe, prolonged, pressure overload during acute admission leading to RV remodelling acutely but some scarring secondary to hypo-perfused myocardium may have occurred leading to persistent RV impairment. Microthrombotic mediated infarction at time of COVID-19 infection may also play a role.

Care must be taken when drawing conclusions on the patients in this follow-up study. By the very nature of follow-up, those who were assessed had declared themselves survivors from COVID-19 infection. They may therefore represent a less severe sample of the hospitalised population. Hence, we compared the demographics, blood biomarkers, comorbid conditions, admission haemodynamics and ventilation status of the follow-up patients with patients who were not followed up but hospitalised for COVID-19. The follow-up group differed in having lower troponin and NT-proBNP as well as less cardiovascular disease and cancer. However, the percentage of patients undergoing ventilation were similar between follow-up vs non-follow-up groups and respiratory manifestations such as oxygen saturations and respiratory rates were worse in the follow-up group. It would therefore appear that the follow-up group represented a healthier population exposed to severe disease. The healthier starting point likely underlies the survivorship bias and leaves the issue of longer term effects of COVID-19 only partially answered. This possibly leads to underestimation of the severity of effect on cardiac function for LV, RV and pulmonary parameters with persistence of significant disease precipitating an earlier death prior to follow-up TTE.

### Limitations

As already pointed out above, this is a study of a particular sample within the hospitalised COVID-19 population, namely survivors of COVID-19. It therefore does not represent the full spectrum of presentation and subsequent natural history of cardiac manifestations of COVID-19. As such, the applicability of the results of the study are limited to survivors of COVID-19. In addition to this, the criteria for referral to TTE were not standardized and therefore a referral bias was possibly introduced.

As with any single centre study, there needs to be caution as to the applicability to the general population. Single centre studies may be subject to various confounders introduced by local population characteristics such as ethnicity, social class, age, gender etc. For example, this study involved more males than females (73% males) and a predominantly (85%) non-white population.

With three individuals conducting and reading echocardiograms and one expert adjudicator, there is the possibility of bias being introduced. No intraobserver or interobserver variability is measured and therefore reliability of interpretation is not tested. Reader specific bias can be introduced possibly compounded by confirmation bias of an unblinded expert adjudicator.

Finally, the advanced parameters of 3D and strain where measured only in the follow-up patients. With no data to directly compare to from admission, it is not possible to determine how much of the abnormality was preexisting, particularly that which was subclinical. The complexity of the interactions of the characteristics (e.g. age, BMI, sex, ethnicity cardiovascular risk factors) of the patients makes it challenging to estimate expected strain values to try to then estimate the effect due to COVID-19 infection. As such this data must be interpreted with caution.

## Conclusion

When considering follow-up (median 17.6 months) of survivors of COVID-19 requiring admission to hospital, there appears to be no significant change in RV or LV dysfunction where present. Rather, there is improvement in RV remodelling with reduction of dilatation and a significant decrease in pulmonary pressures with all markers of pulmonary pressure improving from admission TTE to follow-up TTE. This suggests that as the predominant lung pathology that typifies COVID-19 infection resolves, associated elevated pulmonary pressures also resolve with subsequent improvement in right heart size. However, the impact on RV myocardium remains and is possibly mediated by additional mechanisms to an initial acute severe prolonged pressure overload causing relative ischaemia, including myocarditis, as seen in the left ventricle, which is also affected though at a lower rate, as well as micro-infarction from microthrombi but very little by coronary artery disease.

## Supplementary Information

Below is the link to the electronic supplementary material.

Supplementary file1 (DOCX 20 KB)

## Data Availability

The final anonymous dataset that supports the findings of this study are available from the corresponding author, Roxy Senior, upon reasonable request.
