# Supplementary material for: Follow-up study of the cardiac manifestations of multiethnic patients hospitalised with COVID-19
Source: J Ultrasound. 2026 Jul 30;29(4):805–13. doi: 10.1007/s40477-026-01193-1 (PMC13768518; doi:10.1007/s40477-026-01193-1)
Supplement: Supplementary file 1 [file 40477_2026_1193_MOESM1_ESM.docx]

**Supplementary material**

**Table S1**. Table of recorded variables for demographics, blood biomarkers, comorbid conditions, haemodynamics prior to echocardiogram, and ventilation. Variables for follow-up patients were compared with non-follow-up patients. Means and standard deviations or medians and interquartile ranges are displayed for continuous variables and number and percentage for categorical variables. Number in square bracket records total number of patients. Significant p values are in bold.

| **Variable** | **Follow-up patients (117)** | **Non-follow-up patients (275)** | **P value** |
| --- | --- | --- | --- |
| **Demographics** |  |  |  |
| Age at admission, years, mean (±SD) | 59.4 (±12.8) [117] | 66.8 (±14.7) [275] | **<0.001** |
| Gender, male, number (%) | 85 (73%) [117] | 171 (62%) [275] | **0.046** |
| Ethnicity, white, number (%) | 20 (20%) [102] | 64 (27%) [234] | 0.132 |
| BMI (kg/m²), mean, (±SD) | 29.3 (±6.9) [109] | 27.8 (±7.1) [264] | **0.020** |
| **Blood biomarkers** |  |  |  |
| 1st Troponin T (ng/L) (normal range 0-15), median (IQR) | 17 (32) [97] | 26 (52) [215] | **0.012** |
| 1st NT-proBNP (ng/L), median (IQR) | 377 (1052) [70] | 1237 (4718) [172] | **<0.001** |
| 1st D-dimer (ug/L) (normal range 0 - 500), median (IQR) | 2100 (6931) [91] | 1870 (8870) [193] | 0.877 |
| 1st Ferritin (ug/L) (normal range 30-400), median (IQR) | 953 (1573) [84] | 804 (1067) [195] | 0.252 |
| **Co-morbid conditions** |  |  |  |
| Hypertension, number (%) | 60 (51%) [117] | 154 (56%) [275] | 0.391 |
| Diabetes, number (%) | 43 (37%) [117] | 121 (44%) [275] | 0.183 |
| Cardiovascular disease, number (%) | 17 (15%) [117] | 79 (29%) [275] | **0.003** |
| Cerebrovascular disease, number (%) | 8 (7%) [117] | 36 (13%) [275] | 0.073 |
| Cancer, number (%) | 5 (4%) [117] | 33 (12%) [275] | **0.018** |
| CKD, number (%) | 11 (9%) [117] | 42 (15%) [275] | 0.120 |
| Respiratory disease, number (%) | 28 (24%) [117] | 68 (25%) [275] | 0.867 |
| **Haemodynamics prior to echocardiogram** |  |  |  |
| Admission Heart Rate (beats per minute), mean (±SD) | 101 (±19.9) [73] | 102 (±24.3) [145] | 0.866 |
| Admission Respiratory Rate (breaths per minute), mean (±SD) | 33 (±9.7) [73] | 30 (±9.7) [144] | **0.013** |
| Admission Saturations on air (%), mean (±SD) | 87 (±14.4) [75] | 92 (±7.7) [144] | **0.022** |
| Admission Systolic BP, mmHg, mean (±SD) | 136 (±21.0) [73] | 135 (±25.3) [143] | 0.693 |
| **Ventilation** |  |  |  |
| Ventilated patients, number (%) | 43 (37%) [117] | 110 (40%) [274] | 0.529 |

**Table S2**. Table of recorded variables for left heart, right heart and pulmonary haemodynamics. Variables for early follow-up patients were compared with late follow-up patients. Means and standard deviations are displayed for continuous variables and number and percentage for categorical variables. Number in square bracket records total number of patients. Significant p values are in bold.

| **Variable** | **Early follow-up (59)** | **Late follow-up (58)** | **P value** |
| --- | --- | --- | --- |
| **Left heart parameters** |  |  |  |
| LV end-systolic dimension (cm) mean (±SD) | 3.0 (±0.6) [58] | 2.9 (±0.6) [57] | 0.825 |
| LV end-diastolic dimension (cm) mean (±SD) | 4.6 (±0.6) [59] | 4.5 (±0.6) [58] | 0.311 |
| LA vol index (ml/m²), mean (±SD) | 25.7 (±8.8) [56] | 27.7 (±11.1) [53] | 0.471 |
| E/A, mean (±SD) | 0.8 (±0.3) [56] | 0.9 (±0.3) [55] | 0.126 |
| E/e' average, mean (±SD) | 9.2 (±3.4) [58] | 9.2 (±3.4) [56] | 0.805 |
| E/e' average >14, number (%) | 6 (10%) [58] | 4 (7%) [56] | 0.743 |
| New elevated LV filling pressure + Normal LVEF >50%, number (%) | 4 (7%) [58] | 3 (5%) [56] | 1.000 |
| New LV RWMA, number (%) | 2 (4%) [49] | 3 (5%) [55] | 1.000 |
| Abnormal LVEF (<50%), number (%) | 7 (12%) [59] | 1 (2%) [58] | 0.061 |
| **Right heart parameters** |  |  |  |
| RV impairment, number (%) | 14 (24%) [59] | 19 (33%) [57] | 0.252 |
| TAPSE (cm), mean (±SD) | 2.0 (±0.5) [56] | 2.1 (±0.4) [54] | 0.346 |
| Reduced TAPSE <1.7cm, number (%) | 8 (14%) [56] | 6 (11%) [54] | 0.617 |
| FAC (%), mean (±SD) | 40.2(±7.5) [53] | 39.0(±9.7) [52] | 0.486 |
| Reduced FAC <35%, number (%) | 11 (21%) [53] | 16 (31%) [52] | 0.240 |
| RV S’ TDI (cm/s), mean (±SD) | 13.4(±3.5) [55] | 12.9(±3.0) [55] | 0.487 |
| Basal RV diameter (cm), mean (±SD) | 3.3(±0.6) [59] | 3.3(±0.6) [55] | 0.922 |
| Dilated basal RV >4.1cm, number (%) | 4 (7%) [59] | 3 (5%) [55] | 1.000 |
| Mid RV diameter (cm), mean (±SD) | 2.7(±0.6) [59] | 2.7(±0.5) [55] | 0.506 |
| Dilated mid RV >3.5cm, number (%) | 7 (12%) [59] | 3 (5%) [56] | 0.323 |
| RVOT VTI (cm), mean (±SD) | 15.6 (±4.9) [59] | 14.9 (±3.3) [58] | 0.403 |
| **Pulmonary haemodynamics** |  |  |  |
| D-shaped LV, number (%) | 0 (0%) [58] | 0 (0%) [56] | - |
| PV acceleration time (msec), mean (±SD) | 117.0 (±28.2) [59] | 123.0 (±31.8) [58] | 0.277 |
| PVAT <80ms, number (%) | 4 (7%) [59] | 3 (5%) [58] | 1.000 |
| Pulmonary artery systolic pressure (mmHg), mean (±SD) | 27.6 (±7.3) [14] | 28.3 (±8.4) [20] | 0.823 |
| Pulmonary vascular resistance (Wood units), mean (±SD) | 1.8 (±0.5) [14] | 1.7 (±0.4) [19] | 0.815 |
| Elevated pulmonary pressure, number (%) | 4 (7%) [59] | 4 (7%) [58] | 1.000 |
| RA filling pressure (mmhg), mean (±SD) | 5.1 (±0.7) [54] | 5.1 (±0.4) [56] | 0.969 |
